# Fabrication and vibration characterization of curcumin extracted from turmeric (*Curcuma longa*) rhizomes of the northern Vietnam

**DOI:** 10.1186/s40064-016-2812-2

**Published:** 2016-07-22

**Authors:** Hoang Van Nong, Le Xuan Hung, Pham Nam Thang, Vu Duc Chinh, Le Van Vu, Phan Tien Dung, Tran Van Trung, Pham Thu Nga

**Affiliations:** Institute of Materials Science, Vietnam Academy of Science and Technology (VAST), 18, Hoang Quoc Viet Road, Cau Giay Dist., Hanoi, Vietnam; Institute of Research and Development, Duy Tan University, Danang, Vietnam; Center for Materials Science, University of Natural Science (VNU), Hanoi, Vietnam; Department of Application and Development of Technology, Vietnam Academy of Science and Technology (VAST), Hanoi, Vietnam; Development and Application of Science and Technology Company (DAST), Hanoi, Vietnam

**Keywords:** Curcumin, Crystalline phase, Vibration frequency, Raman spectroscopy

## Abstract

In this report, we present the research results on using the conventional method and microwave technology to extract curcuminoid from turmeric roots originated in different regions of Northern Vietnam. This method is simple, yet economical, non-toxic and still able to achieve high extraction performance to get curcuminoid from turmeric roots. The detailed results on the Raman vibration spectra combined with X-ray powder diffraction and high-performance liquid chromatography/mass spectrometry allowed the evaluation of each batch of curcumin crystalline powder sample received, under the conditions of applied fabrication technology. Also, the absorption and fluorescence spectroscopies of the samples are presented in the paper. The information to be presented in this paper: absorption and fluorescence spectroscopies of the samples; new experimental study results on applied technology to mass-produce curcumin from turmeric rhizomes; comparative study results between fabricated samples and marketing curcumin products—to state the complexity of co-existing crystalline phase in curcumin powder samples. We noticed that, it is possible to use the vibration line at ~959 cm^−1^—characteristic of the ν C=O vibration, and the ~1625 cm^−1^ line—characteristic of the ν C=O and ν C=C vibration in curcumin molecules, for preliminary quality assessment of naturally originated curcumin crystalline powder samples. Data on these new optical spectra will contribute to the bringing of detailed information on natural curcumin in Vietnam, serving research purposes and applications of natural curcumin powder and nanocurcumin in Vietnam, as well as being initial materials for the pharmaceutical, cosmetics or functional food industries.

## Background

Curcumin is a natural, yellow colored phenolic antioxidant and was first extracted in an impure form by Vogel et al. (1815). Curcumin is a hydrophobic phenol having the chemical name [1,7-bis (4-hydroxy-3-methoxyphenyl)-1,6-heptadiene-3,5-dione (Kolev et al. 2005) and empirical formula C_21_H_20_O_6_. Turmeric does not contain only curcumin (compound I), but also its analogues demetoxycurcumin (compound II), bisdemetoxycurcumin (compound III) along with the water-soluble protein turmerin, and curcumin is responsible for its characteristic yellow-to-bright-orange color. The biological and molecular properties of curcumin and its analogues are similar, thus it is suggested that in natural pathways the bisdemethoxycurcumin converts to demethoxycucumin, which in turn converts to curcumin.

Curcumin is known for its wide-ranging pharmacological applications such as antioxidant, anti-inflammatory, antimicrobial, antimalarial, anti-carcinogenic, anti-HIV agent, etc. (Sanphui et al. 2011; Agarwal and Sung 2009). Curcumin, being a diphenolic compound extracted from the rhizome of turmeric, is a prominent candidate for treating cystic fibrosis, Alzheimer’s and malarial diseases in addition to cancer (Maheshwari et al. 2006; Yallapu et al. 2012). Curcumin is safe even at a high dose of 12 g per day (Qureshi et al. 1992; Lao et al. 2006) proven by experiments on both animals and humans. The first crystal structure of curcumin was reported in 1982 in the monoclinic space group P2/n (Tønnesen et al. 1982).

In Vietnam, detailed studies on the crystalline phase and optical spectra of curcumin powder extracted from yellow turmeric are yet to be announced. In this study, we present the research results on natural curcuminoid samples, extracted from turmeric rhizomes grown in Northern Vietnam using the microwave technology, and compare it with the conventional method. The primary purpose of this research is to analyze the technology to produce curcumin, the initial material for producing nanocurcumin, from Vietnamese turmeric rhizomes, and apply it in the pharmaceutical industry. Therefore, we have conducted comparative studies on the crystalline phase of natural curcumin crystal powder, between being fabricated using the conventional method and microwave technology, then present the absorption and fluorescence spectroscopy in this report. This is the first detailed study, carried out on curcumin extracted from Vietnamese turmeric rhizomes with the production scale of hundreds of kilograms of turmeric. The analyzed curcumin content by the high performance liquid chromatography/mass spectrometry method (HPLC/MS) on the three components of curcuminoid was presented to clarify the studies on crystalline phase structure of these natural powder samples.

## Experimental

### Methods

#### Materials

Fresh rhizomes of *Curcuma longa* are purchased from local markets at Khoai Chau, Hung Yen and Hai Duong. After collection, fresh rhizomes were immediately kept in shed, washed with normal water, then again with distilled water. For the next step, they were peeled, shredded into small threads and then ready for curcumin extraction. Different solvents used were absolute ethanol, hexane and isopropanol, all with the pure grade (P.A.), purchased from Xilong Chemical Co. Ltd. (China).

#### Curcumin extracting methods

We used two methods: (1) conventional solvent-extraction method with eco-friendly absolute ethanol, and (2) microwave assisted extraction. These methods were mentioned early in Priyadarsini (2014), Paulucci et al. (2013), Lee et al. (2012a, b), Li et al. (2014), Patel et al. (2000), Kim et al. (2013).

##### Conventional solvent-extraction method

Fiber-formed rhizome (1 kg) is kept in a 3 L amber glass beaker and extracted at room temperature with 1 L of absolute ethanol, soaked and stirred (400 rounds/min). Cover the beaker to prevent loss of ethanol, then leave it for a week. The extracts were filtered separately under vacuum. They can be removed from the ethanol and stored for use as a dry composition. The prepared ethanol extract is transferred to the round-bottomed flask of a rotary evaporator and heated to a maximum temperature of 45 °C. The condensed ethanol is collected via a condenser for the purpose of re-using in subsequent extractions, and the rest of the extracted solution that is free from solvent is removed from the flask for curcumin precipitation with hexane. The resultant powder is stored in an amber bottle until needed. The dried powder was weighed accurately and percentage yield was calculated (sample N1). Afterwards, weigh out a small amount of this sample and prepared it into a solution with concentration 1 mg/mL for HPLC/MS analysis.

##### Microwave assisted extraction

An extraction system comprised of a domestic microwave oven manufactured by SANIO Electric Co. Ltd. equipped with a magnetron of 2450 MHz with a nominal maximum power of 1000 W, 10 power levels, timed controller, convection temperature sensors and exhaust system is used for microwave extraction. The microwave-assisted extraction is carried out as following: place 1 kg of the fresh curcuma threads in a glass vessel and irradiated for a pre-defined time period (1, 3, 5 min) at 200 W microwave power. After irradiation, pour the absolute ethanol into the curcuma longthreads (mass: solvent ratio 1:1 w/v). The ethanol-soaked curcuma longthreads are irradiated at 800 W microwave power for 2, 4 and 6 min. The extraction is performed at 700 W microwave power for 5 min. After the defined extraction period, the samples are collected from the extraction vessel, filtered, and rotary-vacuumed to rid of the solvent, until the volume of the solution remains 30 % compared to the original. Next, take this solution out of the flask, remove the essence and oil from curcumin, then crystallize it with hexane. Finally, the powder sample is weighed and prepared into a 1 mg/mL solution for content analysis using the HPLC/MS method. Hence, we have samples N2, N3, N4, N5, N5-1 and N12, N13.

### Characterization techniques for curcumin

The fabricated curcumin is analyzed with high performance liquid chromatography/mass spectrometry (HPLC/MS) of Agilent 1260 Series Single Quadrupole LC/MS Systems (Agilent Technologies, USA). Stock solutions of curcumin (200 μg/mL) was prepared by dissolving accurately weighed 10 mg of curcumin in methanol using 50 mL volumetric flask. This solution was further diluted with methanol to obtain standard solutions in the concentration range of 1–20 μg/mL. The separation was carried out in a Zorbax Eclipse XDB C18 column (4.6 × 150 mm, 5 µm) with a C_18_ guard column maintained at 24 °C. The elution was performed at flow rate of 0.5 mL/min with a gradient mobile phase from 10 to 30 % acetonitrile in water in 5 min, followed by an increase from 30 to 70 % acetonitrile in another 20 min. The injection volume was of 5 µL. The DAD acquisition wavelength for demethoxycurcumin, bisdemethoxycurcumin and curcumin detection was set at 425 nm. The mass detector used a MM-ES multimode source. OpenLAB software (Agilent Technologies) was employed to control and collect sample. The ion peaks at *m/z* 339.0, 309.1, and 369.1 ([M + H]^+^) were detected for demethoxycurcumin, bisdemethoxycurcumin and curcumin, respectively.

Moreover, the curcumin samples are analyzed by Micro Raman spectroscopy (Explore-Horiba) using 785 nm excitation line from a diode pumped, solid state laser at 100 mW to analyze the vibrational bonds and their Raman frequencies. 10× Objectives are used to focus the excitation laser light on the right spot of the investigated samples, the spot size of the laser beam is 1 μm, spectral resolution is 2 cm^−1^, and acquisition time is always 10 s.

X-ray powder diffraction (XRD) method (Siemens D5005) is used to identify curcumin crystalline phase in the samples. The UV–Vis spectra of curcumin in ethanol are scanned within the wavelength range of 200–600 nm using a Shimadzu (UV-1800) UV–Vis spectrophotometer. All UV–Vis measurements are performed at 25 °C and automatically corrected for the solvent medium, which is the absolute ethanol. The photoluminescence (PL) spectra of curcumin deposited on glass slit are recorded at room temperature on a Fluorolog-3 Model FL3-22 spectrofluorometer system (HORIBA Jobin–Yvon). The emission spectra are measured utilizing a 450 W xenon lamp as the excitation source.

## Results and discussion

### HPLC method for curcumin analysis

**Fig. 1:**
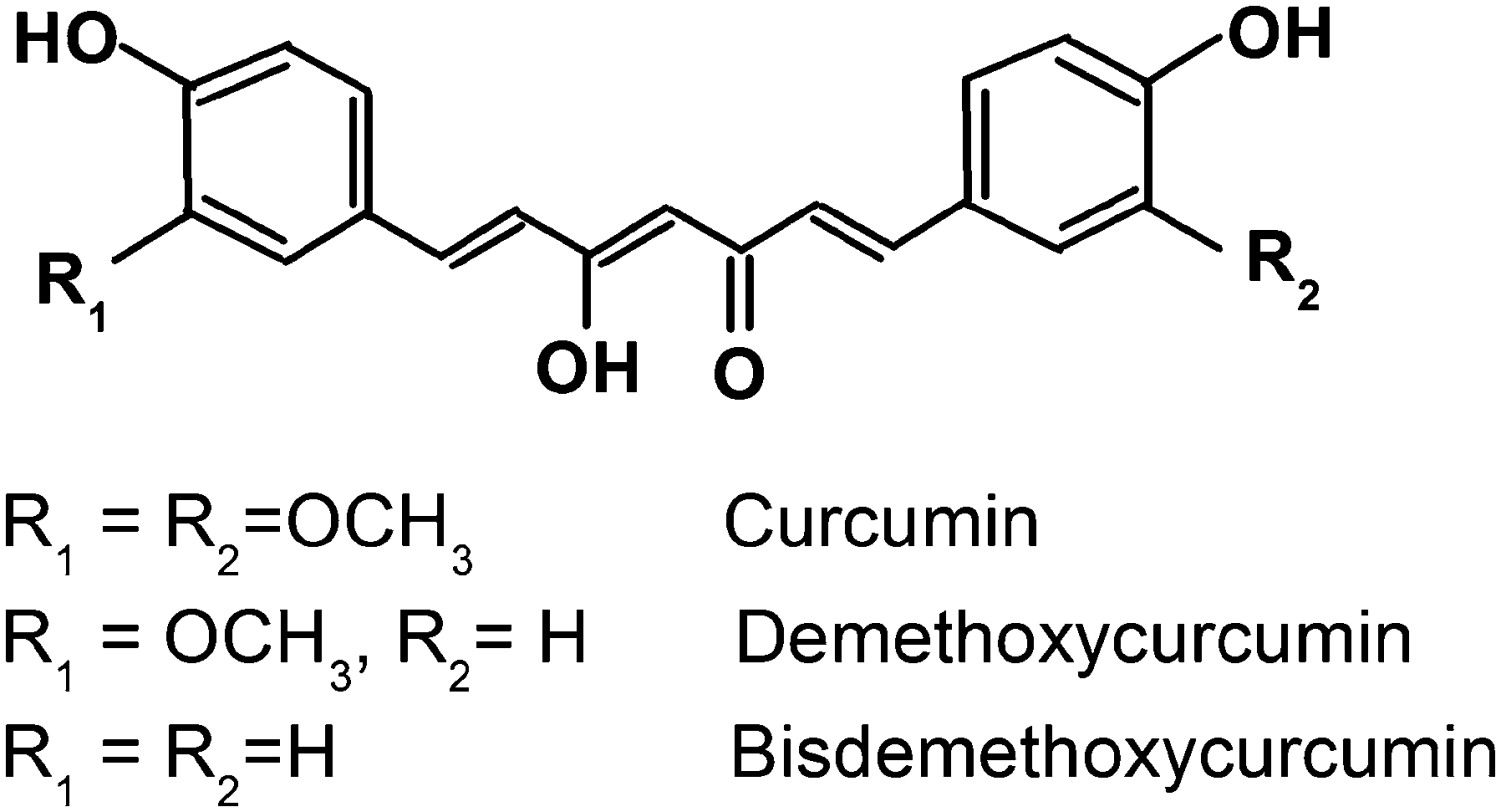
Chemical structure of the curcumin of *Curcuma longa* (Tønnesen 2009)

Curcumin samples are pumped into the chrommatographic system with predefined conditions. Curcumin (I), demethoxycurcumin (II), and bisdemethoxycurumin (III) are three major curcuminoids that coexist in most curcumin products. Therefore, a sensitive HPLC method to separate and quantify the three curcuminoids is established first before. The chemical structure of the curcumin of *Curcuma longa* is presented in Fig. 1.

The peaks of demethoxycurcumin (II), bisdemethoxycurcumin (III) and curcumin (I) are spotted based on the match in the retention time Rt and mass number MS compared to the indicator substance. The mass percentages of the three compounds and content analysis result of the substances are calculated.

We have carried out content analysis of curcumin in some market products, which are named samples N6 and N7 (two samples of the same manufacturer with the same label, and both are nano curcumin according to the company’s instructions), N8 which only contains curcumin and neither demethoxycurcumin (II) nor bisdemethoxycurcumin (III), N9 and N10 which contain a certain amount of form II and form III. The sample N11 is also a commercial nano cucumin product. These data can be used as references to compare. The analysis data by HPLC/MS method shows that the curcumin samples N1 (54 %), N4 (41.66 %), N5-1 (41.43 %) and N12 (56.1 %) have superior quality, and can be compared to those being sold on the market.

### Curcumin characterization by Micro-Raman and X-ray diffraction

Vibration Raman spectrum is a strong tool to define important information on molecular structures of a chemical substance, especially the chemical molecules of a natural product, since they show some vibration modes of some chemical bonds characteristic of the substance itself. On the other hand, if the substance also exists in crystalline powder form, we can also identify the crystalline phase of that substance by the X-ray diffraction (XRD) method. Therefore, we have begun to register micro-Raman spectra and XRD in order to have structural information on curcumin and the crystalline phase of fabricated powder samples under the extracting conditions of room temperature and in a microwave with different power levels and time periods.

#### Raman spectroscopy

**Fig. 2:**
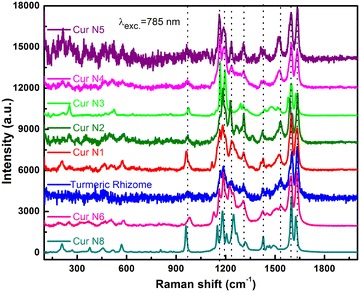
Raman spectra comparison of natural curcumin samples fabricated in this study (N1–N5) with the commercial curcumin samples (N6, N8) and turmeric rhizome in Vietnam

Figure 2 is the Raman spectra of fabricated samples and of fresh turmeric. We can see that most of the observed vibration spectral lines in fresh turmeric also appear in the spectra of all fabricated curcumin samples. This proves that the quality of fabricated samples are high and of natural origins. In order to compare their quality with products on the Vietnamese market, we have recorded the Raman spectra of available products on the market (N6 and N8). Figure 2 presents the comparison of the Raman spectra of these two products along with the ones that we have fabricated. From the spectra of Fig. 2 we can see that most of the vibration lines of the samples nearly overlap. The values of vibration frequencies observed by experiments are also presented in Table 1. The assignment of vibration frequencies of respective bonds are also presented in Table 1.

**Table 1:** Experimental Raman (crystalline powder) spectral data of curcumin in frequency region 1700–900 cm^−1^

Peak assignment	ν	ν	ν	ν	ν	ν	ν_Cur_	ν_Cur_	ν_Cur_	ν	ν	ν_Cal_.
	_Cur N1_	_Cur N2_	_Cur N3_	_Cur N3-1 6 months later_	_Cur N4_	_N6_	_N8_	_N9_	_N10_	Mangolim et al. (2014)	Kolev et al. (2005)	Kolev et al. (2005)
ν C=O (II)		1637			1636					1638		
ν C=O (III)										1639		
ν C=O (I) νC=C	1632		1627	1625		1632	1625	1626	1625	1626	1626	1630
ν C=C (I, II) Aromatic	1599	1599	1599	1599	1598	1599	1599	1600	1599	1600 1602	1601	1615
νC=C (II, III) Aromatic		1590	1579	1579						1591 1591		1587
ν C=O	1523	1536	1516	1523	1524	1536	1531	1533	1529		1509	1536
Phenol C–O (I)	1428		1435			1428	1429	1427	1428	1430	1431	1420
Phenol C–O (II, III)	1413	1413		1413	1413	1413				1416 1415		1409
Enol C–O (I)	1248	1248			1248	1248	1248	1247	1247	1249	1230	1216
Enol C–O (II, III)	1226	1229	1234	1226	1226	1226				1233 1234		1212
			1196				1205	1205	1205		1207	1196
	1183	1183	1168	1183	1183	1187	1181	1182	1182		1184	1176
	1166	1166		1166	1166	1161					1168	1169
	1148	1148		1148	1148		1150	1149	1149			1150
	1118	1118	1120	1118	1118	1128					1120	1107
ν C=O	962.6	971	976	975	975	981	959	961	961		967	966

Vibrational modes: ν stretching

Similar to observations in Fig. 1, we can see that the vibration spectra of fabricated natural product samples from N1 to N4 all possess nearly the same characteristic lines and similar to those recorded from fresh turmeric grown in Northern Vietnam. The vibration spectra of this fresh turmeric are similar to the Raman spectra of those reported before (Bich et al. 2009). The vibration spectra of sample N6 is very similar to those of the fabricated samples and have been presented in this paper. Comparing to the Raman spectrum of the sample N8, which only contains curcumin (I), we see that other than the observed vibration lines at frequencies being the same, there are two lines that are characteristic of curcumin’s vibration frequencies (I) whose peak, in samples containing curcumin (II) and (III), is shifted towards frequencies with larger values. Observed quite clearly, being characteristic of curcumin form I chemical structure, they are lines that appear at frequency 959 cm^−1^—characteristic of vibration ν(C=O) stretching, and at frequency 1625 cm^−1^—characteristic of vibration ν (C=O) stretching or C=C stretching (form I) of curcumin (Kolev et al. 2005; Sanphui et al. 2011; Krishna Mohan et al. 2012). From the detailed analysis of the positions of these two vibration lines, combined with curcumin I, II, III values defined using the HPLC/MS method and the positions of these two peaks on the Raman spectra, it can be concluded—in a quantitative way right after recording the Raman spectrum of a sample—that the sample contains more or less curcumin form I. From comparing positions of frequencies at 959 and 1625 cm^−1^, it clarifies that in the series of samples for production research, sample N1 contains the most curcumin with higher quality compared to the rest. However, sample N6 may contain less curcumin I than samples N8 and N1, despite having the exact same origin as samples fabricated from turmeric in this research (Fig. 2).

**Fig. 3:**
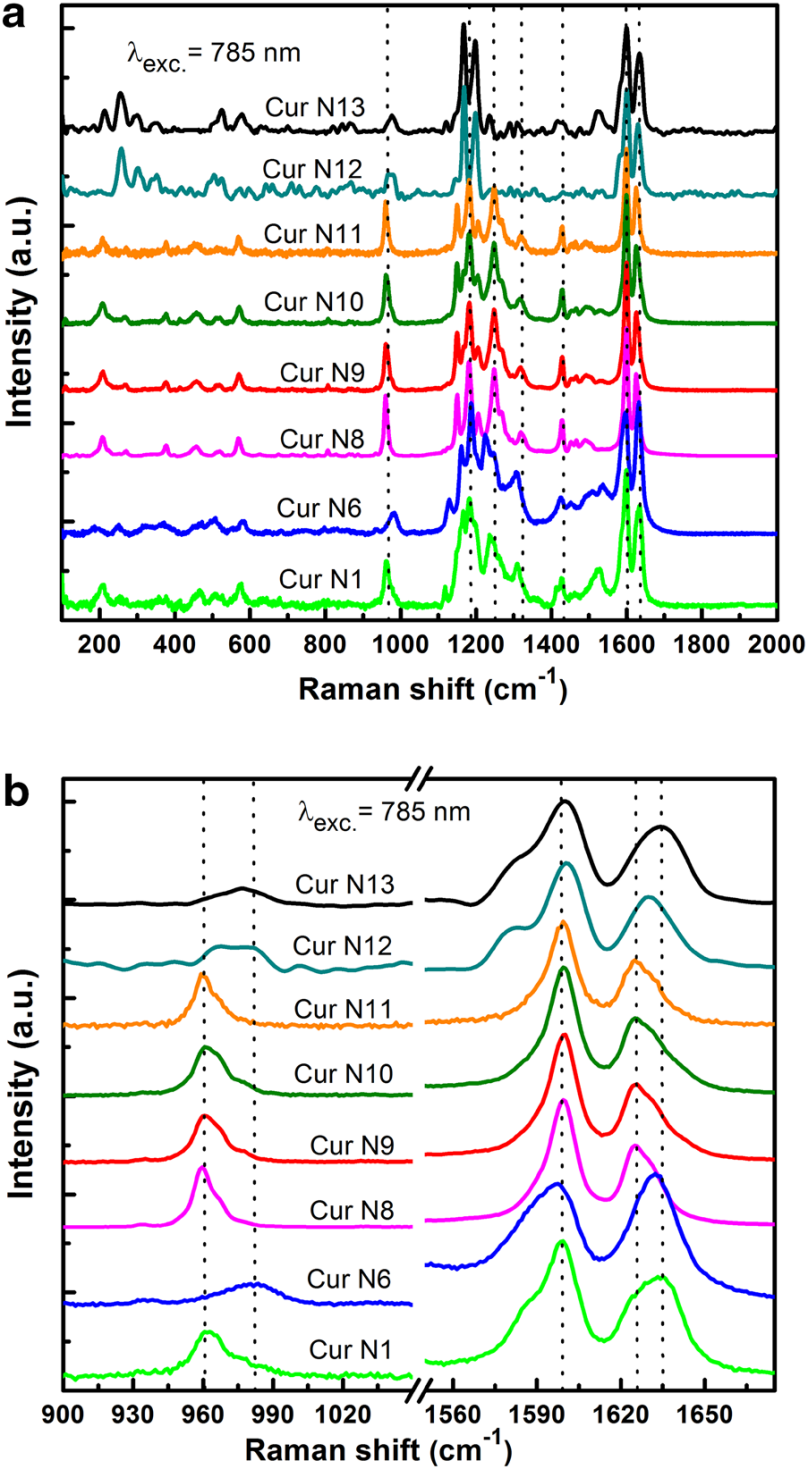
Raman spectra comparison of commercial curcumin samples being sold on the Vietnamese market and fabricated (Cur N1) sample (**a**), and a section of the spectra in the frequency range from 1550 to 1650 cm^−1^ is zoomed in for easy observation of differences at 959 and 1625 cm^−1^ for each different sample, plus samples N12, N13 (**b**)

We have also recorded the Raman spectra of five samples being sold on the Vietnamese market, aiming to study their quality. It can be seen that other than the fact that samples N1, N12, N13 are fabricated samples while N6 is not, the four mentioned samples have very similar spectra (Fig. 3a), being only slightly different at the peaks with frequency 1625 cm^−1^ (Fig. 3b). However, the position of the three samples’ line is shifted approximately 6 cm^−1^ towards longer frequencies. The HPLC/MS content analysis values also show that only N8 curcumin sample contains curcumin I entirely, while the other two contain curcumin I along with its analogues that always form I, which are demetoxycurcumin (II) and bisdemetoxycurcumin (III). In the case of sample N6, the vibration line at 959 cm^−1^—characteristic of curcumin I—is shifted towards frequencies longer than 21 cm^−1^ (Fig. 3b), therefore we can be quite certain that sample N6 has a different origin compared to the other three.

We have also carried out experiments to clean, crystallize and re-crystallize curcumin multiple times with fabricated samples. The Raman measurement results show that the Raman spectra do not change after crystallizations. With the same sample N1 kept for 6 months, we dissolved, re-crystallized and recorded its Raman spectra to find that the Raman spectra of these samples remain completely unchanged, and still the same as those of the original sample N1 and fresh turmeric.

#### X-ray diffraction

**Fig. 4:**
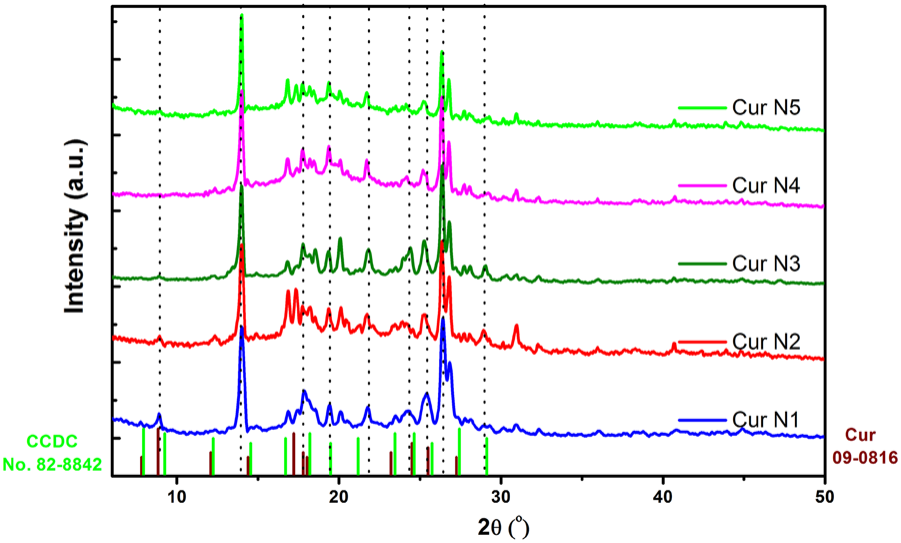
Powder XRD patterns of curcumin samples N1, N2, N3, N4 and N5 with different extraction conditions. Bulk diffraction peaks for curcumin are indexed for identification purpose (JCPDS card 9-816 and CCDC 82-8842)

The fabricated curcumin samples are evaluated using X-ray diffraction (Fig. 4). The X-ray diffraction patterns of all powder samples exhibit a series of thin and intense lines, which are indicative of crystalline. The X-ray diffraction pattern of the sample was the sum of the spectral lines of three components that were present, as expected. It can be observed on the XRD patterns that some lines overlap with those of the curcumin crystalline phase of JCPDS standard card (9-816), and some lines match with the standard card of Cambridge Crystallographic Data Centre (CCDC No. 82-8842), as seen on Fig. 4. Position 2θ of some lines are shifted compared to the lines of standard card 9-816, which is also observed by some other authors (Liu et al. 2014; Gately and Triezenberg 2014; Liu 2013; Singh et al. 2014). It can be seen that these curcumin crystalline powder samples exist two crystal types form II and III, as pointed out from the result of HPLC analysis. As a result, we can observe some lines not matching with standard card 9-816. Therefore, with the XRD method, it can be concluded that the fabricated powder samples are crystallized which are not curcumin I single phase, but can contain congeners and other polymorph crystalline phases. The distinction between forms II and III is some what complicated by the fact that their powder XRD lines are very close (Sanphui et al. 2011). In this research, we are not yet able to afford deeper studies on this matter.

### Absorption and PL spectra of curcumin

**Fig. 5:**
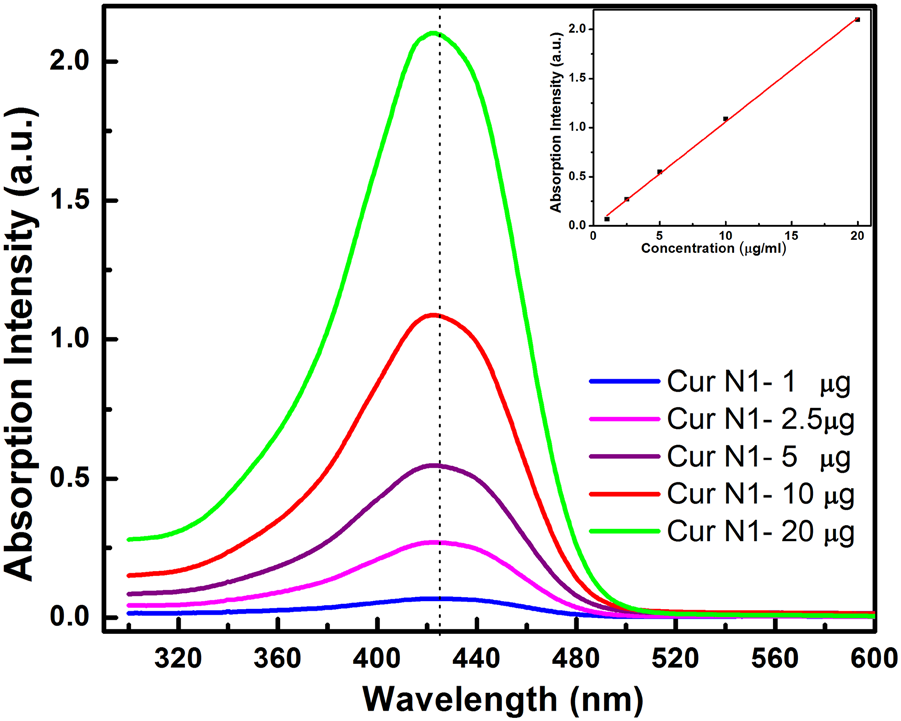
Absorption spectra of curcumin—ethanol solutions with different curcumin concentration from 1, 2.5, 5, 10 μg and 20 μg/mL. The curcumin solution shows absorbance at *λ* = 425 nm and linear increasing absorption at peak 425 nm when the curcumin concentration increased. The *inset* is linear relation of the absorption intensity and curcumin concentration in this concentration range

The UV–Vis spectra of curcumin represent transitions between electronic energy levels. These transitions are generally between a bonding or lone-pair orbital and an unfilled non-bonding or anti-bonding orbital. The absorption spectra of curcumin dissolved in ethanol is presented in Fig. 5 with curcumin concentration varying from 1, 2.5, 5, 10 and 20 μg. The absorption spectrum of curcumin is a broad band with maximum absorbance peak at a wavelength ~425 nm, which could be assigned to low energy π–π* excitation of the curcumin, as reported in Kim et al. (2013). This peak is typical for curcumin dissolved in organic solvent. We see that the more the curcumin solution is diluted, the more the UV–VIS absorption intensity decreases.

The more diluted the curcumin solution is, the more the UV–Vis absorption intensity decreases. The main reason the absorbance decreases in aqueous solution when concentration decreases is the decrease in the number of absorption centers, while on the other hand it is also the degradation of curcumin in water medium by a reaction at the keto-enol group (Singh et al. 2014).

**Fig. 6:**
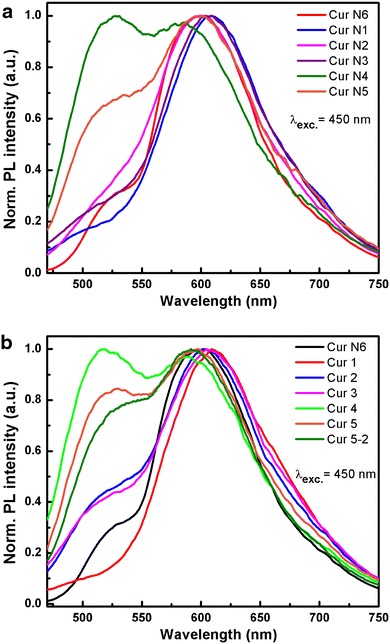
Normalized PL spectra of fabricated curcumin samples in solids form and sample N6 (**a** *upper*) and the same curcumin samples left for 6 months and sample N6 (**b** *bottom*)

**Fig. 7:**
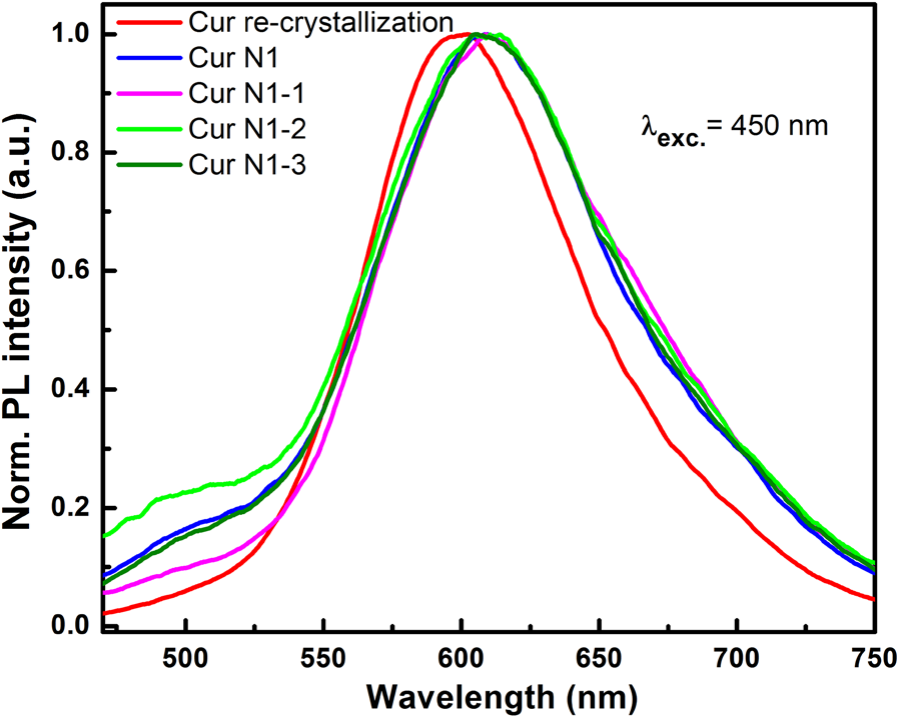
Normalized PL spectra of curcumin sample N1 which is purified and crystallized multiple times, left for 6 months and re-crystallized

Photoluminescence (PL) of the curcumin samples has been recorded for comparison by excitation at the same wavelength of 425 nm in powder form. Figure 6a shows the PL spectra of fabricated curcumin samples and of sample N6. The PL spectrum is a broad emission band, and its maximum peak shifts slightly depending on each sample. The peak of sample N1 is at 607 nm, N3 at 604 nm, while N2, N5 and N6 all have nearly-overlapping peaks at 597 nm. However, sample N5 has a second emission shoulder band at 528 nm and this overlaps with that of N4. Sample N4 also has an emission peak at 586 nm. The (n, π*) character of the carbonyl groups in curcuminoid can affect the shift of the fluorescence maxima. Chignell et al. reported that curcumin fluoresced strongly in toluene (Chignell et al. 1994; Bong 2000), and that the PL intensity as well as the position of the most intense band of curcumin was very sensitive to the nature of the solvent, unlike its absorption maximum. We have also recorded the PL spectrum of sample N6 to consult. The PL spectra of samples kept in the dark for 6 months show no change and no shift of peak happened (Fig. 6b). The comparison with commercial sample N6 sample show no potential differences. For sample N1, when crystallized to purify many times, the spectra do not change. For sample N1 that has been maintained in the dark for 6 months then re-crystallized, the emission peak shifts towards short wavelengths ~10 nm and there are no emission shoulder band at short wavelengths ~500 nm (Fig. 7).

**Fig. 8:**
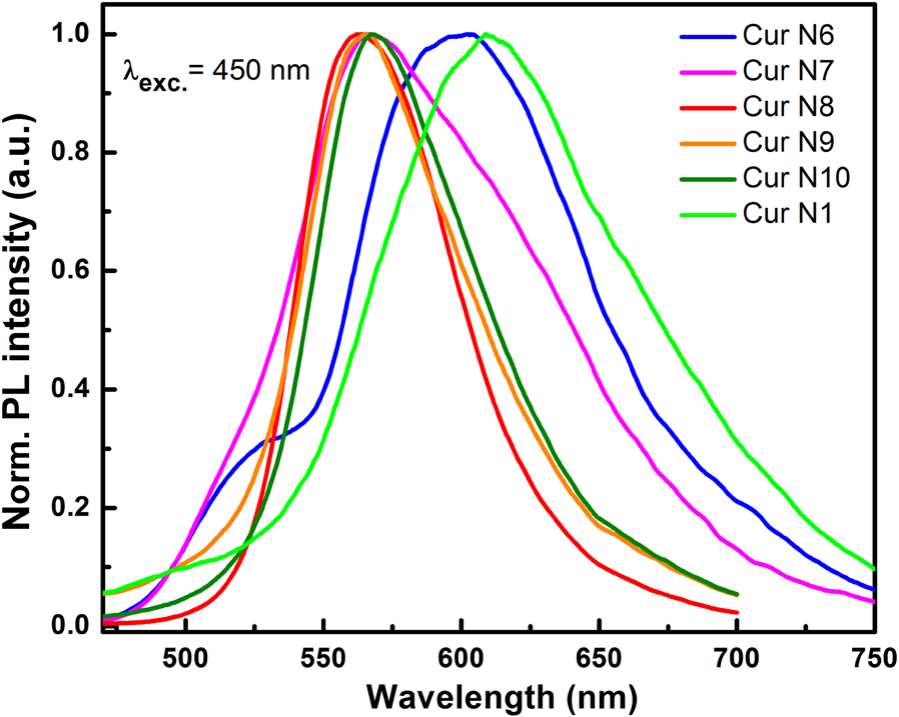
Normalized PL spectra comparison of commercial curcumin samples being sold on the Vietnamese market and sample curcumin N1

Figure 8 is the normalized PL spectra of commercial samples used in this research in comparison with the fabricated curcumin N1. We can see that the PL spectra of sample N1 remain unchanged. The PL spectra of samples N6 and N7 are different, even though their Raman vibration spectra do not change. The PL spectra of the three commercial samples on the Vietnamese market (N8, N9 and N10) all have the same PL spectra, maximum at 567 nm. The emission spectra is a nearly-symmetric band, especially with the curcumin I sample N8. Therefore, we can say that there are significant differences in fluorescent properties between research samples. The system of samples from N1 to N5 has similar emission properties to sample N6. However, with two different samples N6 and N7, the fluorescent spectra are different. This also requires more thoughts. The use of PL spectra also allows the evaluation of quality and stability of the curcumin products. This is an effective evaluation tool in addition to the Raman spectra method to assess product qualities.

## Conclusion

We have succeeded in fabricating a large amount of curcumin from turmeric of northern Vietnam. The extraction method, with the help of microwave technology, saved much extraction time and produced high-quality curcumin which can be compared with the nano curcumin available on the Vietnamese market. Furthermore, the study also allows evaluation of the curcumin sample fabricated by extracting with ethanol at room temperature. This research on Raman and PL spectra allows the curcumin quality evaluation as well as comparison and identification of the fabricated and commercial products on the Vietnamese market. The study of Raman spectra shows that it is possible to use the two vibration lines at frequencies ~959 and ~1625 cm^−1^ to evaluate the quality of the curcumin sample as well as identifying curcumin of natural or synthesis origins.

The research has pointed out that our fabricated curcumin has achieved the first step, which is being made from Vietnamese yellow turmeric (*Curcuma Longa* Linn), has quality that can be compared well to those being sold on the Vietnamese market, and stable after being maintained for 6 months at room temperature. Our fabricated curcumin product serves well for being a natural initial material in nanotechnology to fabricate nanocurcumin used in the pharmaceutical industry, healing burns, cosmetics, functional food and foods.

## References

[CR1] Agarwal BB, Sung B (2009). Pharmacological basis for the role of curcumin in chronic diseases: an age-old spice with modern targets. Trends Pharmacol Sci.

[CR2] Bich VT, Thuy NT, Binh NT, Huong NTM, Yen PND, Luong TT (2009). Structural and spectral properties of curcumin and metal-curcumin complex derived from turmeric (*Curcuma longa*). Phys Eng New Mater.

[CR3] Bong PH (2000). Spectral and photophysical behaviors of curcumin and curcuminoids. Bull Korean Chem Soc.

[CR4] Chignell CF, Bilski P, Reszka KJ, Motten AG, Sik RH, Dahl TA (1994). Spectral and photochemical properties of curcumin. Photochem Photobiol.

[CR5] Gately ST, Triezenberg SJ (2014) Solid forms of curcumin, US Patent, Pub. No. US 2014/0031403

[CR6] Kim YJ, Lee HJ, Shin Y (2013). Optimization and validation of high-performance liquid chromatography method for individual curcuminoids in turmeric by heat-refluxed extraction. J Agric Food Chem.

[CR7] Kim HJ, Kim DJ, Karthick SN, Hemalatha KV, Raj CJ, Ok S, Choe Y (2013). Curcumin dye extracted from *Curcuma longa* L. used as sensitizers for efficient dye-sensitized solar cells. Int J Electrochem Sci.

[CR8] Kolev TM, Velcheva EA, Stamboliyska BA, Spiteller M (2005). DFT and Experimental studies of the structure and vibrational spectra of curcumin. Inter J Quant Chem V.

[CR9] Krishna Mohan PR, Sreelakshmi G, Muraleedharan CV, Joseph R (2012). Water soluble complexes of curcumin with cyclodextrins: characterization by FT-Raman vibrational spectroscopy.

[CR10] Lao CD, Ruffin MT, Normolle D, Heath DD, Murray SI, Bailey JM, Boggs ME, Crowell J, Rock CL, Brenner DE (2006). Dose escalation of a curcuminoid formulation. BMC Complement Altern Med..

[CR11] Lee KJ, Yang HJ, Jeong SW, Ma JY (2012). Solid-phase extraction of curcuminoid from turmeric using physical process method. Korean J Pharmacogn.

[CR12] Lee KJ, Ma JY, Kim YS, Kim DS, Jin Y (2012). High purity extraction and simultaneous high-performance liquid chromatography analysis of curcuminoids in turmeric. J Appl Biol Chem.

[CR13] Li M, Ngadi MO, Ma Y (2014). Optimisation of pulsed ultrasonic and microwave-assisted extraction for curcuminoids by response surface methodology and kinetic study. Food Chem.

[CR14] Liu D (2013) Engineering nano-curcumin with enhanced solubility and in vitro anti-cancer bioactivity. Master Thesis of the State University of New Jersey

[CR15] Liu J, Chen F, Tian W, Ma Y, Li J, Zhao G (2014). Optimization and characterization of curcumin loaded in octenylsuccinate oat β-glucan micelles with an emphasis on degree of substitution and molecular weight. J Agric Food Chem.

[CR16] Maheshwari RK, Singh AK, Gaddipati J, Srimal RC (2006). Multiple biological activities of curcumin: a short review. Life Sci.

[CR17] Mangolim CS, Moriwaki C, Nogueira AC, Sato F, Baessoc ML, Neto AM, Matioli G (2014). Curcumin–β-cyclodextrin inclusion complex: stability, solubility, characterisation by FT-IR, FT-Raman, X-ray diffraction and photoacoustic spectroscopy, and food application. Food Chem.

[CR18] Patel K, Krishna G, Sokoloski E, Ito Y (2000). Preparative separation of curcuminoids from crude curcumin and turmeric powder by pH-zone-refining counter current chromatography. J Liquid Chromatogr.

[CR19] Paulucci VP, Couto RO, Teixeira CCC, Freitas LAP (2013). Optimization of the extraction of curcumin from *Curcuma longa* rhizomes. Braz J Pharmacogn.

[CR20] Priyadarsini KI (2014). The chemistry of curcumin: from extraction to therapeutic agent. Molecules.

[CR21] Qureshi S, Shah AH, Ageel AM (1992). Toxicity studies on *Alpinia galanga* and *Curcuma longa*. Planta Med.

[CR22] Sanphui P, Goud NR, Khandavilli UBR, Bhanoth S, Nangia A (2011). New polymorphs of curcumin. Chem Commun.

[CR23] Singh PK, Wani K, Ghanekar RK, Prabhune A, Ogale S (2014). From micron to nano-curcumin by sophorolipid co-processing: highly enhanced bioavailability, fluorescence, and anti-cancer efficacy. RSC Adv.

[CR24] Tønnesen HH (2009). Phenolic compounds in foods and their effects on health I. ACS Sym Series.

[CR25] Tønnesen HH, Karlsen J, Mostad A (1982). Structural studies of curcuminoids. I. The crystal structure of curcumin. Acta Chem Scand B.

[CR26] Vogel HA, Pelletier J, Pharmacol J (1815). Curcumin—biological and medicinal properties. J Pharma.

[CR27] Yallapu MM, Jaggi M, Chauhan SC (2012). Curcumin nanoformulations: a future nanomedicine for cancer. Drug Disc Today.

